# Pelvic floor rehabilitation management in females with stress urinary incontinence: a summary of best-evidence strategies

**DOI:** 10.1186/s12912-026-04874-8

**Published:** 2026-06-21

**Authors:** Ping Xu, Xuefen Xu, Hongyan Wang, Xiaojuan Wang, Pingping Guo, Wei Zhang, Minna Mao, Rujia Zhao, Suwen Feng

**Affiliations:** 1 School of Nursing, Shandong Second Medical University, Weifang, Shandong China; 2 Women’s Hospital, Zhejiang University School of Medicine, No. 1 Xue Shi Road, Hangzhou, 310006 China

**Keywords:** Stress urinary incontinence, Pelvic floor muscle training, Lifestyle management, Evidence-based nursing, Evidence summary

## Abstract

**Background:**

The current evidence on pelvic floor rehabilitation management for females with stress urinary incontinence (SUI) is diverse and complex, and standardized pelvic floor rehabilitation protocols are lacking. The study aimed to retrieve and summarize the evidence related to pelvic floor rehabilitation management in females who experience SUI and provide guidance for healthcare workers.

**Methods:**

Following the 6S evidence resource pyramid model, two researchers searched the computer decision support system, guidelines networks, urological professional society websites, obstetrics and gynecology professional society websites, and related databases from top to bottom and collected the best clinical practice information books, clinical decision-making, recommendation practice, evidence summaries, guidelines, systematic reviews, and expert consensuses on the management of pelvic floor rehabilitation in females suffering SUI. The searches were carried out from database inception to September 2022. Literature quality was independently assessed by two researchers, and evidence was then obtained and summarized from eligible studies.

**Results:**

Forty-eight studies were eligible to be included, comprising 1 best clinical practice information book, 4 clinical decision-making, 3 recommended practices, 10 evidence summaries, 8 guidelines, 18 systematic reviews, and 4 expert consensus studies. Best-evidence strategies summarized the evidence for pelvic floor rehabilitation management in females with SUI, which included a total of 51 recommendations in 5 aspects: screening and assessment, lifestyle management, pelvic floor muscle training (PFMT), follow-up, and health education.

**Conclusion:**

The review summarized 51 best-evidence strategies for pelvic floor rehabilitation management in females with SUI. To ensure clinical efficacy and improve nursing quality, these strategies should be integrated into structured, multidisciplinary clinical pathways, carefully tailored to specific patient contexts and local healthcare resources.

**Supplementary Information:**

The online version contains supplementary material available at https://doi.org/10.1186/s12912-026-04874-8.

## Background

Urinary incontinence (UI), defined as a condition of involuntary loss of urine, is a prevalent urological problem in women [1]. Stress urinary incontinence (SUI), the most common type of UI among females [2], is characterized by urinary leakage during physical exertion, coughing, and sneezing [3]. A study has shown that the prevalence of SUI may be as high as 49%, depending on the population and the method of defining SUI [4]. Female SUI has a negative effect on patients’ physical and mental health as well as their social relationships.

Initial treatment of SUI begins with non-invasive and low-cost conservative treatment consisting primarily of pelvic floor muscle training (PFMT) and lifestyle modification [5]. Among them, lifestyle modification includes fluid management, regular physical activity, avoidance of constipation, weight management, and smoking cessation [6, 7]. PFMT and lifestyle interventions are important components of pelvic floor rehabilitation, which can reduce UI symptoms and improve physical condition [8–10].

The effectiveness of PFMT and lifestyle modification may vary depending on the treatment regimen implemented. Several high-quality studies have been generated on the conservative management of UI to guide clinical practice [2, 11]. However, the available evidence on the management of pelvic floor rehabilitation for women with SUI is varied and complex; for example, there are extensive training programs available for PFMT, but a standardized PFMT program is lacking [12]. Studies to date have not reached a consensus on the content and considerations for standardized pelvic floor rehabilitation management for females with SUI. The lack of standardized protocols, variability in clinical practice, and inadequate information may lead to organizational variation in women’s health services, thereby worsening the accessibility and acceptability of pelvic floor rehabilitation management services.

Evidence-based health care interventions are particularly important for promoting pelvic floor rehabilitation in females with SUI. In the study, we used an evidence-based medicine approach to summarize the best evidence for pelvic floor rehabilitation management in females who experience SUI by searching existing studies on pelvic floor rehabilitation management in females with SUI, with the aim of providing a reference for healthcare professionals when administering related pelvic floor rehabilitation care.

## Methods

### Search strategy

Following the 6S evidence resource pyramid model [13], two researchers searched the computer decision support system, guideline networks, urological professional society websites, obstetrics and gynecology professional society websites, and related databases in order from top to bottom, including (1) the computer decision support system: BMJ Best Practice, Up To Date. (2) Guideline networks: Guidelines International Network (GIN), National Institute for Health and Care Excellence (NICE), Australian Clinical Practice Guidelines (ACPG), Scottish Intercollegiate Guidelines Network (SIGN), New Zealand Guidelines Group (NZGG), Registered Nurses’ Association of Ontario (RNAO), and Emmetone. (3) Urological professional society websites: American Urological Association (AUA), International Gynecologic Association (IUGA), American Gynecologic Society (AUGS), Canadian Urological Association (CUA), European Association of Urology (EAU), Society of Urodynamics, Female Pelvic Medicine and Urogenital Reconstruction (SUFU), European Society of Urogenital Radiology (ESUR), British Society of Obstetrics and Gynaecology (BSUG), Australian and New Zealand Association of Urology (USANZ), British Association of Urological Surgeons (BAUS), International Society of Continence (ICS). (4) Obstetrics and gynecology professional society websites: American College of Obstetricians and Gynecologists (ACOG), Royal Australian and New Zealand College of Obstetricians and Gynaecologists (RANZCOG), Society of Obstetricians and Gynecologists of Canada (SOGC), Royal College of Obstetricians and Gynaecologists (RCOG), and Society of Obstetric Medicine of Australia and New Zealand (SOMANZ). (5) Related databases: Joanna Briggs Institute (JBI), Cochrane Library, Web of Science, PubMed, CINAHL, Embase, PEDro, Scopus, CNKI, CBM, WanFang Data, VIP, and the Chinese Medical Association full-text journal database.

Search terms “urinary incontinence” and “pelvic floor disorders” were used to retrieve relevant evidence from the computer decision support system, guideline networks, and professional society websites. MeSH terms and keywords were applied to retrieve relevant evidence in the database. MeSH and keywords used included: “urinary incontinence, stress”, “urinary leakage”, “pelvic floor muscle training”, “lifestyle intervention*”, “self-management”, “guideline*”, “evidence summary”, “consensus*”, “systematic review”, “meta-analysis”, etc. A literature search was conducted from database inception to September 2022. Taking PubMed as an example, a detailed retrieval strategy is available in Additional File 1.

### Inclusion and exclusion criteria

The inclusion criteria were as follows: (i) Chinese or English were the languages of publication; (ii) the study population was women with SUI; (iii) the content was related to pelvic floor rehabilitation management of patients with SUI, including assessment, health education, PFMT, lifestyle interventions (such as exercise management, weight management, and constipation prevention), and follow-up; (iv) the type of evidence included best clinical practice information books, clinical decision-making, recommendation practice, evidence summaries, guidelines, systematic reviews, and expert consensuses. The exclusion criteria included: (i) direct translation or duplication of literature; (ii) unavailability of full text; (iii) interpreted literature, brief versions of guidelines, and literature that is not the latest edition; (iv) low-quality literature; (v) content related to urge urinary incontinence, mixed urinary incontinence, and neurological urinary incontinence; (vi) literature that is not within the last 5 years.

### Literature screening

Two researchers independently read titles, abstracts, and full-text articles according to the inclusion and exclusion criteria, gradually screening the studies. Disagreements were resolved through consultation with a third researcher.

### Quality assessment

The categories of best practice, clinical decision-making, recommendation practice, and evidence summary are the higher-level types of evidence in the evidence pyramid; however, there are no internationally accepted tools for evaluating the quality of the literature for these categories of evidence; therefore, for this type of study: (1) directly identify evidence from authoritative databases (e.g., BMJ Best Practice, UpToDate, and JBI Evidence-Based Center databases) as high-quality evidence for this type of evidence; (2) for other sources of evidence in this category, evaluate whether the process of evidence formation has strictly followed the standards of evidence development and the corresponding process, and if not, judge it as low-quality evidence and exclude it from inclusion.

The Appraisal of Guidelines for Research & Evaluation II (AGREE II) was used to evaluate the guidelines’ methodological quality [14].

Corresponding appraisal tools from the JBI evidence-based health care centers in Australia were used to evaluate the quality of systematic reviews and expert consensuses [15].

The quality assessment was completed independently by two researchers who had systematically studied evidence-based nursing, and in case of disagreement, the final decision was made by a third person who had evidence-based research training experience.

### Evidence extraction and synthesis

#### Evidence extraction

According to the research questions, two researchers read the incorporated literature piece by piece and independently extracted the content related to pelvic floor rehabilitation management in females suffering from SUI. The evidence was extracted by two researchers proficient in foreign languages who independently translated the relevant evidence and compared the results, and if there were any problems, they were agreed upon through discussion. An Excel sheet was used to extract information, including first author/developing organization, title, source of evidence, date of publication, and type of evidence.

### Evidence classification and integration

After the evidence extraction was completed, the evidence was classified and integrated. When conflicting evidence was identified across sources, prioritization was determined systematically: (1) Evidence-based evidence priority: evidence from rigorous systematic reviews or evidence syntheses was favoured over lower-level evidence such as expert consensus; (2) Higher quality evidence priority: among studies of similar design, those with higher methodological quality were given priority; (3) Most recent authoritative literature priority: when the level and quality of evidence were comparable, the most recently published literature from authoritative sources was selected. Any residual conflicts were resolved through team discussion. The evidence level classification utilized the JBI Evidence Pre-grading System (2014 edition, Table 1) [16]. The level of evidence was divided into levels 1–5 according to the design category of the included study. If the same evidence entry originated from different types of original studies/multiple bodies of evidence of different levels, it was pre-graded according to the highest quality evidence.

**Table 1:** The JBI evidence pre-grading system (2014 edition) [16]

Level of Evidence	Examples of Study Design	Description
Level 1	Experimental Designs	1a: Systematic review of Randomized Controlled Trials (RCTs)
		1b: Systematic review of RCTs and other study designs
		1c: Randomized Controlled Trial (RCT)
		1d: Pseudo-RCT
Level 2	Quasi-experimental Designs	2a: Systematic review of quasi-experimental studies
		2b: Systematic review of quasi-experimental and other lower study designs
		2c: Quasi-experimental prospectively controlled study
		2d: Pre-test and post-test or historic/retrospective control group study
Level 3	Observational - Analytic Designs	3a: Systematic review of comparable cohort studies
		3b: Systematic review of comparable cohort and other lower study designs
		3c: Cohort study with control group
		3d: Case-controlled study
		3e: Observational study without a control group
Level 4	Observational - Descriptive Designs	4a: Systematic review of descriptive studies
		4b: Cross-sectional study
		4c: Case series
		4d: Case study
Level 5	Expert Opinion / Bench Research	5a: Systematic review of expert opinion
		5b: Expert consensus
		5c: Bench research / single expert opinion

## Results

### Literature selection and the basic characteristics

**Fig. 1:**
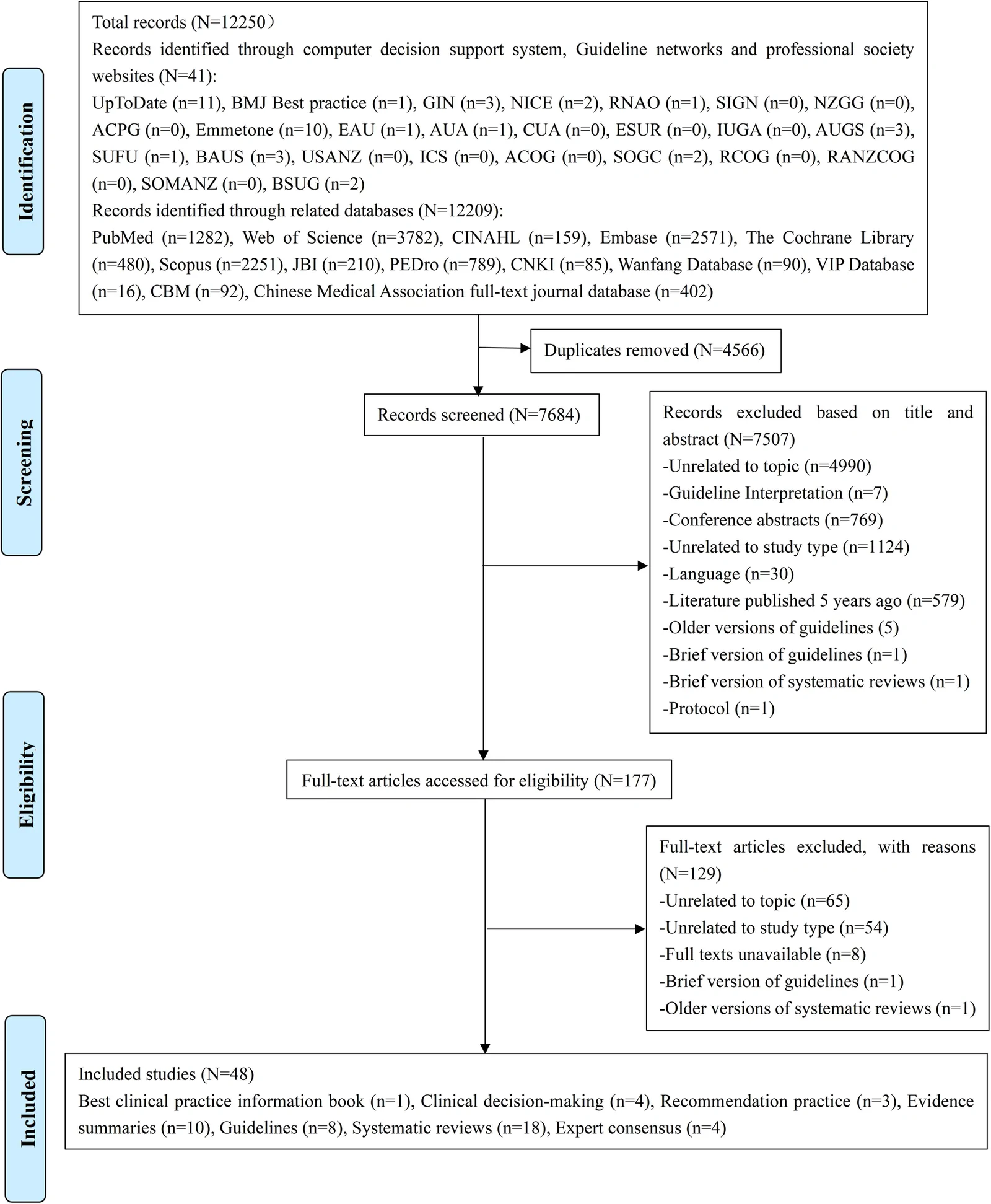
Evidence retrieval and screening process

In total, 12,250 records were obtained through preliminary searches, including 41 records from computer decision support systems, guideline networks, and related professional society websites, and 12,209 records from related databases. Among them, 4566 records were removed from duplicate literature through EndNote literature management software and manual screening, and 7,636 records were excluded after reading titles, abstracts, and full texts. Finally, 48 studies were included in this study, including 1 best practice [17], 4 clinical decision-making [18–21], 3 recommended practices [22–24], 10 evidence summaries [25–34], 8 guidelines [35–42], 18 systematic reviews [43–60], and 4 expert consensus studies [61–64]. The screening process for literature is displayed in Fig. 1.

Table 2 summarizes the basic information about the included studies. The included studies were published between 2017 and 2022, of which 9 studies [34, 43–46, 55, 60, 62, 63] were published in Chinese, and the remaining were reported in English.

**Table 2:** Basic characteristics of included studies

No.	First author/developing organization (year)	Title	Source of evidence	Type of evidence
1	Davila (2020) [17]	Urinary incontinence in women	BMJ Best Practice	Best clinical practice information book
2	Lukacz (2022) [18]	Female urinary incontinence: evaluation	UpToDate	Clinical decision-making
3	Lukacz (2022) [19]	Female urinary incontinence: treatment	UpToDate	Clinical decision-making
4	Crowley (2022) [20]	Patient education: urinary incontinence in females (the basics)	UpToDate	Clinical decision-making
5	Lukacz (2022) [21]	Patient education: urinary incontinence treatments for women (beyond the basics)	UpToDate	Clinical decision-making
6	JBI (2021) [22]	Urinary incontinence (older people): management by personal care workers	JBI	Recommendation practice
7	JBI (2022) [23]	Urinary incontinence in women: prenatal pelvic floor muscle training	JBI	Recommendation practice
8	JBI (2021) [24]	Urinary incontinence: conservative management	JBI	Recommendation practice
9	Jayasekara (2022) [25]	Urinary incontinence: prompted voiding	JBI	Evidence summary
10	Moola (2022) [26]	Stress and urge urinary incontinence in women: pelvic floor muscle training	JBI	Evidence summary
11	Koh (2022) [27]	Urinary incontinence: timed voiding	JBI	Evidence summary
12	Porritt (2021) [28]	Pelvic floor muscle training (antenatal and postnatal): prevention and treatment of urinary incontinence	JBI	Evidence summary
13	Mathew (2021) [29]	Urinary incontinence: treatments	JBI	Evidence summary
14	Magtoto (2021) [30]	Urinary incontinence: conservative management	JBI	Evidence summary
15	Aginga (2021) [31]	Urinary incontinence: assessment	JBI	Evidence summary
16	Lizarondo (2021) [32]	Urinary incontinence in older adults: pelvic floor muscle training	JBI	Evidence summary
17	Fong (2021) [33]	Urinary incontinence (older person): assessment	JBI	Evidence summary
18	Wang (2022) [34]	Summary of the best evidence for self-management in patients with urinary incontinence	CNKI	Evidence summary
19	Harding (2022) [35]	Management of non-neurogenic female lower urinary tract symptoms (LUTS)	EAU	Guideline
20	Registered Nurses’ Association of Ontario (2020) [36]	A proactive approach to bladder and bowel management in adults	RNAO	Guideline
21	Dufour (2020) [37]	No. 397 − Conservative care of urinary incontinence in women	SOGC	Guideline
22	National Institute for Health and Care Excellence (2021) [38]	pelvic floor dysfunction: prevention and non-surgical management	NICE	Guideline
23	National Institute for Health and Care Excellence (2019) [39]	Urinary incontinence and pelvic organ prolapse in women: management	NICE	Guideline
24	Stangel-Wojcikiewicz (2021) [40]	Urogynecology Section of the Polish Society of Gynecologists and Obstetricians guidelines on the management of stress urinary incontinence in women	Emmetone	Guideline
25	Abrams (2017) [41]	Evaluation and treatment of urinary incontinence, pelvic organ prolapse and faecal incontinence	UpToDate	Guideline
26	Rawin (2019) [42]	Urinary incontinence	UpToDate	Guideline
27	Gao (2020) [43]	Systematic assessment on health education of pelvic floor muscle exercise for female patients with stress urinary incontinence	CNKI	Systematic review
28	Xia (2020) [44]	Meta analysis of pelvic floor muscle training for improving postpartum stress urinary incontinence	CNKI	Systematic review
29	Chen (2020) [45]	Meta-analysis of the short- and medium-term efficacy of pelvic floor muscle training to improve urinary incontinence symptoms in women	Chinese Medical Association full-text journal database	Systematic review
30	Wang (2019) [46]	Meta-analysis of effect of pelvic floor muscle training during pregnancy to prevent or treat urinary incontinence in primipara	CNKI	Systematic review
31	Dumoulin (2018) [47]	Pelvic floor muscle training versus no treatment, or inactive control treatments, for urinary incontinence in women	Embase	Systematic review
32	Radzimińska (2018) [48]	The impact of pelvic floor muscle training on the quality of life of women with urinary incontinence: a systematic literature review	Embase	Systematic review
33	Nipa (2020) [49]	Effectiveness of therapeutic interventions for women with urinary incontinence: a systematic review	Embase	Systematic review
34	García-Sánchez (2019) [50]	What pelvic floor muscle training load is optimal in minimizing urine loss in women with stress urinary incontinence? A systematic review and meta-analysis	Embase	Systematic review
35	Nie (2017) [51]	A meta-analysis of pelvic floor muscle training for the treatment of urinary incontinence	Embase	Systematic review
36	Mazur-Bialy (2020) [52]	Urinary incontinence in women: modern methods of physiotherapy as a support for surgical treatment or independent therapy	Scopus	Systematic review
37	Lopez-Liria (2019) [53]	Effectiveness of physiotherapy treatment for urinary incontinence in women: a systematic review	Web of Science	Systematic review
38	Paiva (2017) [54]	Pelvic floor muscle training in groups versus individual or home treatment of women with urinary incontinence: systematic review and meta-analysis	Web of Science	Systematic review
39	Yang (2022) [55]	The effectiveness of group-based pelvic floor muscle training in preventing and treating urinary incontinence for antenatal and postnatal women: a systematic review	Embase	Systematic review
40	Malinauskas (2022) [56]	Efficacy of pelvic floor physiotherapy intervention for stress urinary incontinence in postmenopausal women: systematic review	PubMed	Systematic review
41	Todhunter-Brown (2022) [57]	Conservative interventions for treating urinary incontinence in women: an overview of cochrane systematic reviews	Scopus	Systematic review
42	Wieland (2019) [58]	Yoga for treating urinary incontinence in women	Embase	Systematic review
43	Woodley (2020) [59]	Pelvic floor muscle training for preventing and treating urinary and faecal incontinence in antenatal and postnatal women	Web of Science	Systematic review
44	Zhang (2021) [60]	Meta analysis of the effect of self-management intervention in elderly patients with urinary incontinence	CNKI	Systematic review
45	Bordeianou (2020) [61]	Measuring pelvic floor disorder symptoms using patient-reported instruments	AUGS	Expert consensus
46	Song (2020) [62]	Chinese expert consensus on weight management in obese female patients with stress urinary incontinence (2020)	Emmetone	Expert consensus
47	Gynecological Pelvic Floor Group of the Obstetrics and Gynecology Branch of the Chinese Medical Association (2017) [63]	Guidelines for the diagnosis and treatment of female stress urinary incontinence	Chinese Medical Association full-text journal database	Expert consensus
48	Russo (2021) [64]	Management of urinary incontinence in postmenopausal women: an EMAS clinical guide	Embase	Expert consensus

### Quality assessment results of the included studies

#### Quality assessment of the best practice

One best practice from BMJ Best Practice was included as high-quality evidence [17].

### Quality assessment of the included clinical decision-making studies

Four clinical decision-making studies from UpToDate were included as high-quality evidence [18–21].

### Quality assessment of the included recommended practices

Three recommended practices from the Joanna Briggs Institute were included as high-quality evidence [22–24].

### Quality assessment of the included evidence summaries

As for the ten evidence summaries [25–34], nine studies [25–33] of them were from the JBI Evidence-Based Center database, were of high quality, and were included. Based on the six guidelines and the six systematic reviews, the evidence development process for one study [34] in CNKI basically followed the relevant processes and standards developed for evidence summaries, and it was considered higher-quality evidence and was included in this evidence synthesis.

### Quality assessment of the included guidelines

The methodological quality of the eight included guidelines was evaluated using AGREE II [35–42], as shown in Table 3. Four guidelines [35, 36, 38, 39] had standardized scores > 60% in all domains, which were A-level recommendations and could be directly applied. Three guidelines [37, 40, 41] were B-level recommendations, which could be applied after appropriate modifications. One guideline [42] was a C-level recommendation of poor quality and was excluded.

**Table 3:** Quality assessment of the included guidelines

First author/developing organization (year)	Standardization score of each domain (%)						≥60% of the number of domains	≥30%of the number of domains	The level of recommendation
	Scope and objects	Stakeholder involvement	Rigor of development	Clarity of presentation	Applicability	Editorial independence			
Harding (2022) [35]	100.0	100	82.3	100	95.8	91.7	6	0	A
Registered Nurses’ Association of Ontario (2020) [36]	100.0	100	90.6	100	95.8	100	6	0	A
Dufour (2020) [37]	100.0	47.2	62.5	100	64.6	50	4	0	B
National Institute for Health and Care Excellence (2021) [38]	100.0	97.2	88.5	91.7	100	87.5	6	0	A
National Institute for Health and Care Excellence (2019) [39]	100.0	91.7	100	100	100	91.7	6	0	A
Stangel-Wojcikiewicz (2021) [40]	63.9	33.3	29.2	77.8	35.4	50	2	1	B
Abrams (2017) [41]	80.6	52.8	72.9	100	100	41.7	4	0	B
Rawin (2019) [42]	8.3	11.1	8.3	77.8	41.7	16.7	1	4	C

Note: If the standardized scores of all 6 domains are ≥60%, the recommendation level is A, which means strongly recommended; if the number of domains with standardized scores > 30% is ≥3, but there are <60% domains, the recommendation level is B, which means recommended; if the standardized scores of ≥ 3 domains are <30%, the recommendation level is C, which means not recommended

### Quality assessment of the included systematic reviews

Among the 18 systematic reviews included in this study [43–60], all items of 4 studies [46, 47, 50, 58] were evaluated as “yes” with a complete research design and high overall quality, and were included. Of the four studies [44, 54, 57, 59], all items were “yes” except for one item that was assessed as “unclear” or “no”. For example, Todhunter-Brown et al. [57] only included literature from The Cochrane Library, so its item 4 (“Adequacy of databases or resources for searching literature”) was evaluated as “no”. The four studies were included after an overall evaluation. Six studies were qualitative systematic reviews [43, 48, 49, 52, 53, 56], so item 8 (“Appropriateness of the methodology for combining studies”) was evaluated as “not applicable”. Among them, Radzimińska et al. [48] did not define inclusion criteria according to PICO, so their item 2 (“Appropriateness of the inclusion criteria for this evidence-based question”) was evaluated as “no”. The study by Mazur-Bialy et al. [52] only searched PubMed as a database, so its item 4 (“Whether the databases or resources used to search the literature are adequate”) was evaluated as “no”. In addition, since no quality assessment was conducted, the quality of the study by Mazur-Bialy et al. [52] was evaluated as “not applicable” in item 5 (“Whether the criteria used to evaluate the quality of the literature are appropriate”) and item 6 (“Whether the evaluation of the quality of the literature is done independently by two or more evaluators”). The literature by Mazur-Bialy et al. [52] was excluded after discussion among the research team members because of its low quality. The quality assessment results of systematic reviews are displayed in Table 4.

**Table 4:** Quality assessment of the included systematic reviews

Evaluation items	1	2	3	4	5	6	7	8	9	10	11
Gao (2020) [43]	Y	Y	Y	Y	Y	Y	Y	NA	N	Y	Y
Xia (2020) [44]	Y	Y	Y	Y	Y	Y	U	Y	Y	Y	Y
Chen (2020) [45]	Y	U	Y	Y	Y	U	Y	Y	Y	Y	Y
Wang (2019) [46]	Y	Y	Y	Y	Y	Y	Y	Y	Y	Y	Y
Dumoulin (2018) [47]	Y	Y	Y	Y	Y	Y	Y	Y	Y	Y	Y
Radzimińska (2018) [48]	Y	N	Y	Y	Y	Y	Y	NA	N	Y	Y
Nipa (2020) [49]	Y	Y	U	Y	Y	Y	U	NA	N	Y	Y
García-Sánchez (2019) [50]	Y	Y	Y	Y	Y	Y	Y	Y	Y	Y	Y
Nie (2017) [51]	Y	Y	Y	Y	Y	U	Y	Y	N	Y	Y
Mazur-Bialy (2020) [52]	Y	Y	Y	N	NA	NA	U	NA	N	Y	Y
Lopez-Liria (2019) [53]	Y	Y	Y	Y	Y	U	U	NA	N	Y	Y
Paiva (2017) [54]	Y	Y	Y	Y	Y	Y	Y	Y	N	Y	Y
Yang (2022) [55]	Y	Y	Y	Y	Y	Y	U	Y	N	Y	Y
Malinauskas (2022) [56]	Y	Y	Y	Y	Y	Y	U	NA	N	Y	Y
Todhunter-Brown (2022) [57]	Y	Y	Y	N	Y	Y	Y	Y	Y	Y	Y
Wieland (2019) [58]	Y	Y	Y	Y	Y	Y	Y	Y	Y	Y	Y
Woodley (2020) [59]	Y	Y	Y	Y	Y	Y	Y	Y	N	Y	Y
Zhang (2021) [60]	Y	Y	Y	Y	Y	U	Y	Y	N	Y	Y

Note: Y: Yes, N: No; U: Unclear; NA: Not applicable. 1. whether the proposed evidence-based question is clear and unambiguous; 2. whether the literature inclusion criteria are appropriate for the evidence-based question; 3. whether the search strategy is appropriate; 4. whether the databases or resources used to search the literature are adequate; 5. whether the criteria used to evaluate the quality of the literature are appropriate; 6. whether the evaluation of the quality of the literature is done independently by two or more evaluators; 7. whether certain measures are taken to reduce errors when extracting information; 8. whether the methods used to combine studies are appropriate; 9. whether the recommendations for policy or practice are based on the results of the systematic evaluation; 11. whether the proposed directions for further research are appropriate. Whether the method of combining studies was appropriate; 9. Whether the possibility of publication bias was assessed; 10. Whether the proposed recommendations for policy or practice were based on the results of the systematic evaluation; and 11. Whether the proposed directions for further research were appropriate

### Quality assessment of the included expert consensuses

There were four expert consensuses included in this study [61–64]. Among them, all six evaluation items by the Gynecological Pelvic Floor Group of the Obstetrics and Gynecology Branch of the Chinese Medical Association [63] were “yes”, and the literature was of high quality and was included. For the studies by Bordeianou et al. [62] and Song et al. [61], all evaluation items were rated as “yes” except for item 6 (“Is there any inconsistency between the proposed views and previous literature?”), which was evaluated as “unclear.” However, given their comprehensive content and sound design, both studies were included. In Russo et al. [64], we found a potential conflict of interest regarding item 3 (“Are the proposed views centered on the target population’s interests?”), as some experts received payments from pharmaceutical companies. Consequently, this article was excluded after discussion among the study group. Details are displayed in Table 5.

**Table 5:** Quality assessment of the included expert consensuses

Evaluation items	1	2	3	4	5	6	Whether to include
Bordeianou (2020) [61]	Y	Y	Y	Y	Y	U	Y
Song (2020) [62]	Y	Y	Y	Y	Y	U	Y
Gynecological Pelvic Floor Group of the Obstetrics and Gynecology Branch of the Chinese Medical Association (2017) [63]	Y	Y	Y	Y	Y	Y	Y
Russo (2021) [64]	Y	Y	N	Y	Y	U	N

Note: Y: Yes, N: No; U: Unclear. 1. Whether the source of the point is clearly presented; 2. Whether the views come from influential experts in the field; 3. Whether the proposed views are centered on the interests of the population related to the research; 4. Are the stated conclusions based on the results of the analysis? Are the ideas expressed in a logical manner; 5. Are other existing studies referenced and accurately cited; 6. Is there any inconsistency between the views proposed and previous literature

## Summary of the main recommendations

By summarizing the evidence of pelvic floor rehabilitation management for females with SUI, we formed 51 best evidence strategies from 5 aspects, including screening and assessment, lifestyle management, PFMT, follow-up, and health education, as shown in Table 6. Table 7 lists the core PFMT prescription parameters.

**Table 6:** Best evidence synthesis for pelvic floor rehabilitation management in women with SUI

Evidence item		Evidence content	Evidence level
Screening and assessment	History taking	1. History-taking should include identification of the type of UI (i.e., stress, urge, or mixed), precipitating factors, duration of symptoms, fluid intake (including caffeine and alcohol), perineal skin condition, use of UI products such as pads, the severity of symptoms, and their impact on quality of life [19, 36, 37, 39, 41].	1a
	Objective examination	2. Women are encouraged to complete a voiding diary (work and rest status) for at least 3 days to accurately record the time, volume, duration of leakage, and type of UI [19, 31, 39, 62, 63].	1a
		3. A 1-hour pad test can be performed to quantify leakage if available, but routine use of pad tests should be avoided in women with UI [39, 62, 63].	1a
	Subjective scoring of clinical symptoms	4. Subjective scoring of clinical symptoms can be done using the Ingelman-Sundberg scale. Mild: UI occurs during coughing and sneezing and does not require the use of pads; moderate: UI occurs during daily activities such as running and jumping, fast walking, etc., and requires the use of pads; severe: UI occurs with light activity and changes in lying position [62].	5b
	Assessment tools	5. Validated and appropriate tools can be used as part of a standardized assessment of patients with SUI [35].	1a
		6. Assessment of symptom severity and quality of life: assessment tools are recommended for use with ICIQ-UI-SF, UDI-6, IIQ-7, and I-QOL [18, 19, 31, 33, 43, 59, 61–63].	1a
Pelvic floor muscle training	Exercise prescriptions	7. Advise the patient to place two fingers in the vagina and contract the vaginal muscles by squeezing the fingers from all around to find the correct muscle group [17].	5b
		8. Inform the patient that the abdominal, leg, and hip muscles should remain relaxed while contracting the pelvic floor muscles [17].	5b
		9. Inform patients that they can choose lying, kneeling, or standing positions when performing PFMT [23].	5b
		10. Teach the patient to combine slow contraction training with fast contraction training [50].	1a
		11. Sessions: perform slow contractions for 5–10 seconds and fast contractions for 1–3 seconds. Follow each contraction with a 1–12 second relaxation period. Perform a maximum of nine contractions per set, with a 1–3 minute rest between sets [50].	1a
		12. It is recommended to keep exercise sessions short, lasting 10 to 45 minutes per session, three to seven times per week [50].	1a
		13. Healthcare providers should offer a supervised PFMT program lasting 6 weeks to 6 months for women with SUI, including pregnant women [19, 30, 32, 34, 36, 38–40, 48, 50, 63].	1a
	Guidance and supervision	14. The PFMT program should be guided and supervised by a healthcare professional or physiotherapist with expertise in PFMT [37, 38, 44].	1a
		15. Patients should be instructed on the correct methods of pelvic floor muscle training, the importance of precautions and consistent exercises, etc [43].	1a
		16. The intensity of pelvic floor muscle exercises increases with the number and duration of contractions. Healthcare professionals need to develop a targeted rehabilitation exercise program based on the patient’s condition, and the intensity of training should follow the principle of gradual progression [50].	1a
		17. Women should be informed that they should receive PFMT in the prenatal period [28].	1a
		18. Supervised PFMT (group or one-on-one) is recommended for patients lacking motivation or struggling to contract pelvic floor muscles correctly [19].	1c
		19. Intensive, high-intensity, and supervised PFMT programs, or those that incorporate behavioral interventions or adherence strategies, are of greater benefit to patients with UI [35, 57].	1a
		20. It is recommended to combine supervised PFMT with at-home PFMT [32].	1c
		21. Inform patients that if they suddenly want to urinate during daily life, they can perform three quick pelvic floor contractions to help temporarily control the urge to urinate [37].	5b
		22. If the PFMT program is beneficial, women are advised to continue PFMT after the supervised program [38].	1a
	Monitoring	23. Provide a review to assess the patient’s progress during the pelvic floor rehabilitation program at least once when the provider provides the PFMT program and once at the end of the program [38].	1a
		24. An outpatient follow-up visit should be performed after 3 months of training for the evaluation of subjective and objective treatment outcomes [63].	5b
Lifestyle management	Weight management	25. Assessment and monitoring of body weight [38].	1a
		26. High intra-abdominal pressure in obese women may weaken the supporting structures of the pelvic floor, and a sustained increase in intra-abdominal pressure may also lead to instability of the detrusor muscles, which may precipitate UI. Weight loss is recommended for overweight and obese women with SUI [19, 35, 37, 57].	1a
		27. Diets are not recommended during pregnancy because they may harm the health of the unborn child [38].	1a
		28. Base meals on starchy foods, such as potatoes and rice, and choose whole grains whenever possible [38].	1a
		29. Eat fiber-rich foods such as oats, beans, fruits, and vegetables, as well as whole-grain bread, brown rice, and pasta [38].	1a
		30. Eat at least five servings a day of a variety of fruits and vegetables instead of foods high in fat and calories [38].	1a
		31. Eat a low-fat diet to avoid increasing fat and/or calorie intake [38].	1a
		32. Limit the intake of fried foods and foods high in fat and sugar (e.g., some takeout, fast food, cakes, and pastries) [38].	1a
	Elimination of bladder irritants	33. Alcoholic, caffeinated beverages, carbonated beverages, spicy foods, or acidic foods can irritate the bladder. Teach patients to reduce their intake of foods or beverages that tend to exacerbate symptoms [19, 20, 37].	3c
	Fluid intake management	34. Women are advised to consume small amounts of fluid (usually water) several times throughout the day rather than large amounts of fluid at one time [19, 37].	5b
		35. High fluid intake increases urinary leakage, whereas excessive restriction of fluid intake results in concentrated urine, irritating the bladder and increasing the urgency to urinate. Therefore, patients with UI who have a high or low fluid intake are advised to adjust their fluid intake [19, 37, 38].	1a
		36. If urination is frequent at night, it is recommended to stop or reduce fluid intake two to four hours before bedtime [20, 37, 40].	5b
	Gut management	37. Constipation can worsen UI and increase urinary retention risk. To prevent constipation, patients should consume about 30 grams of dietary fiber daily [19, 37].	5b
		38. Avoiding or limiting chronic straining during toileting may improve UI [57].	1a
	Smoking cessation	39. A smoking-related cough may increase intra-abdominal pressure and exacerbate symptoms of UI, and smokers are advised to quit [17, 57].	1a
	Physical activity	40. Regular physical activity has a positive effect on the pelvic floor muscles and may reduce the frequency of incontinence episodes [40].	5b
		41. Women with UI are encouraged to engage in low- to moderate-intensity physical activity, such as walking, low-impact aerobics, light resistance training, yoga, Pilates, and tai chi [36, 57, 62].	1a
		42. Activities that increase abdominal pressure should be reduced or avoided, as should extra-load resistance training (e.g., heavy lifting), high-intensity jumping (e.g., trampoline), and strenuous exercise such as high-intensity running [40, 57, 63].	1a
		43. Minimize sedentary activities, such as sitting for long periods to watch television, use a computer, or play video games [38].	1a
		44. Integrate activities into daily life, such as walking instead of cycling, cycling instead of motorcycling, walking at lunchtime, etc [38, 62].	1a
	Timed urination	45. Regular urination every 3–4 hours to avoid stress leaks during physical activity with a full bladder [37].	5b
	Skin management	46. Clean the skin immediately after an episode of incontinence to prevent skin breakdown [24].	5b
		47. The use of personal hygiene products, such as pads and sanitary napkins, is recommended to keep the skin dry and to control urine odor [37, 40].	5b
Follow-up	Regular follow-up	48. It is recommended that patients be followed up regularly by their doctors to observe changes in their symptoms and to provide ongoing guidance on PFMT and behavior modification [62].	5b
	Long-term follow-up	49. Long-term follow-up is encouraged. Long-term follow-up (at least one year of follow-up) is necessary if treatment aims to determine the long-term effects of the treatment strategy on UI [47, 49].	1a
Health education	Education content	50. Educate patients about pelvic floor anatomy, causes, risk factors, pathogenesis, and treatment of UI [43, 57].	1a
	Education format	51. Health education implemented through the Internet or smartphones (mobile apps or videos) helps to improve the correctness and adherence of PFMT in patients [19, 43, 49].	1a

**Table 7:** Summary of PFMT core prescription parameters

Parameter Category	Core Recommendations
Position	lying, kneeling, or standing positions are all acceptable [23].
Contraction technique	Alternate between slow contractions (lasting 5–10 seconds) and fast contractions (lasting 1–3 seconds), followed by 1–12 seconds of relaxation. No more than 9 sets per session, with a 1–3 minute rest between sets [50].
Duration per session	10–45 minutes [50].
Frequency	3 to 7 times per week [50].
Total program duration	6 weeks to 6 months [19, 30, 32, 34, 36, 38–40, 48, 50, 63].
Guidance and supervision	Requires guidance and supervision by healthcare professionals [37, 38, 44]. A combined model of supervised training and home-based training is routinely recommended [32]. For patients with poor adherence or difficulty isolating the correct muscles, one-on-one or group-based supervised training is recommended [19].
Principle of progression	Follow the principle of gradual progression. Healthcare professionals should progressively increase the number of contractions, duration, and intensity based on the patient’s specific condition [50].

## Discussion

Referencing high-quality evidence is crucial for providing quality healthcare [65]. The use of scientifically evidence-based interventions for the management of SUI implies the integration of evidence-based knowledge into clinical care practice, which will have a beneficial impact on the effectiveness and quality of care. This study will enable more women with SUI to benefit from evidence-based pelvic floor management.

### Screening and assessment

Evidence 1–6 summarizes the subjective and objective tests and assessment tools used for screening and assessment of patients with SUI, among others. Although some studies have found better adherence with 24-hour voiding diaries [18], we found more studies using 3-day voiding diaries to assess incontinence treatment outcomes [39, 62]. Short, validated questionnaires can also be used to assess the severity of UI and its impact on quality of life. Studies have highlighted that the ICIQ-UI-SF and the UDI-6 are considered the two best tools for scoring UI symptoms [61]. Among these, screening for the type of incontinence and the level of symptom distress can be assessed by the ICIQ-UI-SF scale [61]. The ICIQ-UI-SF scale has been proven to be closely related to 1-hour and 24-hour urine pad tests for assessing the severity of SUI. In addition, the summary score given by the UDI-6 has been shown to correlate with the quality of life index [61].

### PFMT

Evidence 7–24 summarizes the recommendations related to PFMT in patients with SUI, with evidence 7–13 for exercise prescriptions, 14–22 for guidance and supervision, and 23–24 for monitoring patients for PFMT.

Previous studies have shown that women who receive regular (such as weekly) supervision during PFMT are more likely to show improvement than those who receive little or no supervision [66]. The effectiveness of various PFMT methods for treating female UI has been compared in several studies. Paiva et al. explored the effectiveness of individually supervised, group-supervised, and unsupervised home PFMT in treating UI [54]. The research has observed that there is no significant difference between group-based supervised PFMT and individual-based supervised PFMT in UI treatment. However, group-supervised interventions for UI were superior to home PFMT [54]. A systematic review indicated that patients who used a combination of group PFMT and individual PFMT supervision were more likely to report symptom improvement compared to women who received only individual PFMT supervision [66]. Furthermore, one study noted that individual PFMT and group PFMT were similarly effective in improving SUI [67], and no significant differences were found between group and individual therapy in improving patients’ urinary and psychological symptoms and quality of life [68]. Although women may feel embarrassed or intimidated in group therapy [68], group therapy reduces health care and human resource costs compared to individual therapy [54]. Studies have noted that the successful implementation of all PFMT programs, whether individual or group therapy, depends on the therapist’s supervision during the exercises and the patient’s ability to contract the pelvic floor muscle groups correctly [54]. Therefore, providing individual and group training and treatment for women with SUI under professional supervision is important to promote their pelvic floor rehabilitation.

There is no clear consensus in research regarding the choice of parameters related to PFMT, such as intensity and frequency [19, 23, 39, 63]. Referring to the NICE guideline [39], the three included studies recommended a regimen of at least 8 contractions, 3 times a day [30, 32, 34]. Nevertheless, a recent systematic review based on randomized controlled trials clarified variables such as the specific number of contractions for PFMT [50]. Based on the principles of evidence classification and integration, we selected the most recent evidence to present relevant parameters, such as PFMT intensity. One included article showed more effective benefits of high-intensity, high-frequency PFMT compared to low-intensity, low-frequency PFMT [57]. However, it is important to note that PFMT protocols are progressive and individualized [42], and patients should be taught to start with low intensity and low frequency. Another noteworthy aspect is the duration of PFMT. Several studies have recommended that PFMT be maintained for at least 3 months [32, 34, 36, 38, 39, 63]. Some studies have also emphasized that the duration of PFMT should not be shorter than six weeks [48, 50]. Clinical decision-making recommends initial treatment, such as six weeks of PFMT, and up to six months of conservative treatment after progress is made [19]. Furthermore, the optimal timing of PFMT for patients with maternal UI is not fully understood. Some studies have reported that PFMT is advocated for pregnant women in mid- to late pregnancy, considering the possible negative effects of excessive exercise in early pregnancy on embryonic implantation as well as growth and development [69]. One clinical decision-making suggests that the time of initiation of PFMT should be determined according to the mode of delivery [70]. This study stated that exercise could be started in the first or second week after delivery if the woman had not had an episiotomy or perineal tear during vaginal delivery and that PFMT could be started by the third week after delivery if the woman had delivered by cesarean section, as long as there was no pain during contraction of the pelvic floor muscles.

### Lifestyle management

Evidence 25–47 summarizes the recommendations related to lifestyle management for patients with SUI. Lifestyle interventions include weight loss, reduction of bladder irritants such as caffeine intake, fluid intake management, constipation relief, smoking cessation, physical activity management, regular urination, and skin management. Lifestyle management is critical for relieving symptoms of SUI in females. A two-arm randomized controlled trial found that self-monitoring techniques (including fluid and caffeine intake, constipation management, and individualized counseling for voiding frequency) not only improved symptoms but also improved the quality of life of patients with UI [71]. Studies have noted that the benefits of lifestyle management in patients with UI are usually observed within 4–6 weeks [11].

Regarding the relationship between caffeine intake and the development of UI, studies have shown that higher caffeine intake is associated with an increased prevalence of UI [72, 73]. For women with any SUI, the included evidence supports a reduction in caffeine intake. For fluid intake, there is evidence that limiting fluid intake below the recommended daily level (1400 to 1900 mL [approximately 48 to 64 oz]) may alleviate incontinence symptoms [10]. Two studies [2, 74] noted that fluid intake, primarily water, is appropriate at no more than 2000 mL [approximately 68 oz] per day. Rather than drinking large amounts of fluid in a short period of time (e.g., >700 mL [approximately 24 oz] in 30 minutes), strategies for fluid intake advocate frequent intake of small amounts of fluid (e.g., 120 to 150 mL [approximately 4 to 5 oz] per hour) [2, 11].

Other lifestyle changes, such as weight loss and exercise, also promote symptomatic improvement in patients with SUI. Studies have demonstrated an association between obesity and UI [35, 37]. According to the results of a prospective longitudinal study, a 5% weight loss improved the severity of UI and its impact on the quality of life in obese women [75]. A randomized controlled trial that included overweight and obese women with UI found that women who underwent a 6-month intensive weight loss program had a mean weight loss and fewer episodes of SUI compared to patients who received an educational program [76]. This result further suggests that moderate weight loss in overweight or obese women may also improve SUI [76]. In addition, moderate exercise may also improve SUI symptoms. Although strenuous, high-impact activities can trigger UI, low-intensity physical activity is associated with a reduced risk of SUI, according to [77]. Pelvic floor muscles were positively impacted by regular physical activity, which may be associated with exercise to promote weight loss [78].

Although some studies have concluded that the evidence is inadequate to support or refute the use of timed voiding to manage UI [29], recent evidence suggests that certain women who only experience SUI when their bladder capacity is high would benefit from timed voiding because of the ability to keep their bladder capacity lower than it was when SUI occurred [19]. Therefore, encouraging patients to urinate regularly may reduce the frequency of urine leakage in patients with SUI.

### Follow-up

Evidence 48–49 summarize relevant evidence regarding the content of follow-up from 1 expert consensus [62] and 2 systematic reviews [47, 49]. Regular follow-up is essential to maintain patient adherence, observe changes in incontinence symptoms, and assess treatment efficacy. Based on the synthesized evidence, a structured follow-up schedule is recommended: an initial assessment at 3 months post-PFMT instruction to evaluate adherence and early outcomes; a subsequent review at 6 months if treatment goals are only partially met; and an annual long-term follow-up for women who have achieved their goals. This ensures symptom recurrence is monitored while allowing for a personalized follow-up plan tailored to individual needs and local healthcare resources. However, studies have noted that while long-term follow-up is crucial to properly assess intervention effectiveness and evidence reliability [59], current systematic reviews report a distinct lack of long-term follow-up data, particularly concerning quality of life [57]. Therefore, future studies should conduct robust long-term follow-ups to further validate the sustained effects of pelvic floor rehabilitation interventions.

### Health education

Evidence 50–51 summarize relevant evidence regarding health education for patients with SUI from one clinical decision-making [19] and three systematic reviews [43, 49, 57]. Perera et al. reported that despite the high prevalence of SUI, this population has poor healthcare-seeking behavior, with only 12.9% of women with symptoms of UI seeking medical help from healthcare providers [79]. Poor knowledge and misconceptions about UI are considered the greatest barriers to seeking medical care [80]. For example, many people believe that SUI is a normal consequence of aging and pregnancy in women and do not consider SUI to be a disease [79, 81]. Overall, these findings suggest that women have limited knowledge of UI and that a lack of education negatively affects their ability to seek care. Increased knowledge of health issues can be effective in promoting behavior change and improving treatment adherence. Therefore, healthcare providers should pay attention to promoting evidence-based knowledge related to UI, teaching women with SUI about pelvic floor anatomy, pathogenesis, and treatment, as well as tailoring health messages based on different age groups and characteristics (e.g., pregnant women, women in labor, perimenopausal women, and postmenopausal women) [38], which will improve patients understanding of the condition, thereby enhancing adherence, and help women manage symptoms of urinary leakage.

E-health tools such as the Internet and mobile applications are crucial sources of information for females with SUI and can help improve patient self-management and adherence to pelvic floor rehabilitation behaviors. However, studies have pointed out that the quality of e-health-related information varies [41], and most current apps are not related to credible sources or scientific evidence [82]. Therefore, future application development should focus on improving overall quality and incorporating evidence-based content to standardize women’s pelvic floor rehabilitation nursing practice. 

### Limitations

This study has several limitations that should be acknowledged. First, the literature search was restricted to published studies in English and Chinese, which may introduce language bias and limit the global comprehensiveness of the synthesized evidence. Second, as noted in the findings, there is considerable heterogeneity among the included studies regarding specific intervention parameters (such as PFMT frequency and intensity) and patient demographics. Finally, given the significant geographical, cultural, and resource differences across healthcare institutions globally, these summarized strategies cannot be adopted mechanically. Future application of this evidence must be carefully tailored to the specific clinical context, local healthcare resources, and cultural background to develop viable, localized practice plans.

## Conclusion

This study summarized and evaluated 51 best-evidence strategies across five domains—screening and assessment, lifestyle management, PFMT, follow-up, and health education—for pelvic floor rehabilitation in females with SUI. These recommendations provide practical guidance for healthcare providers. To effectively translate this predominantly international evidence into localized clinical practice, we propose a three-step implementation framework: (1) Contextualized Adaptation: Clinicians must tailor these strategies to account for demographic variations (e.g., ethnicity and physiological characteristics), cultural values, and individual patient preferences. (2) Multidisciplinary Integration: The recommendations should be incorporated into structured clinical pathways, serving as assessment checklists and practice guides for collaborative teams (e.g., nurses, physiotherapists, and urogynecologists). (3) System Optimization: Institutions should embed these guidelines into local protocols or electronic health record (EHR)-based decision support tools. Furthermore, recognizing the complexities of evidence translation, future initiatives must conduct systematic analyses of implementation barriers to formulate targeted change strategies.

## Electronic supplementary material

Below is the link to the electronic supplementary material.


Supplementary Material 1


## Data Availability

No datasets were generated or analysed during the current study.

## Abbreviations

UI: Urinary Incontinence
SUI: Stress Urinary Incontinence
PFMT: Pelvic Floor Muscle Training
JBI: Joanna Briggs Institute

## References

[1] Wu JM. Stress incontinence in women. N Engl J Med. 2021;384(25):2428–36. 10.1056/NEJMcp1914037. 34161707

[2] Lukacz ES, Santiago-Lastra Y, Albo ME, Brubaker L. Urinary incontinence in women: a review. JAMA. 2017;318(16):1592–604. 10.1001/jama.2017.12137. 29067433

[3] D’Ancona C, Haylen B, Oelke M, Abranches-Monteiro L, Arnold E, Goldman H, et al. The International Continence society (ICS) report on the terminology for adult male lower urinary tract and pelvic floor symptoms and dysfunction. Neurourol Urodynamics. 2019;38(2):433–77. 10.1002/nau.23897. 30681183

[4] Liu Z, Liu Y, Xu H, He L, Chen Y, Fu L, et al. Effect of electroacupuncture on urinary leakage among women with stress urinary incontinence: a randomized clinical trial. JAMA. 2017;317(24):2493–501. 10.1001/jama.2017.7220. 28655016 PMC5815072

[5] Dumoulin C, Adewuyi T, Booth J, Bradley C, Burgio K, Hagen S, et al. Adult conservative management. In: Incontinence: 6th international consultation on incontinence. Tokyo; 2016 September.

[6] Bo K, Frawley HC, Haylen BT, Abramov Y, Almeida FG, Berghmans B, et al. An International Urogynecological Association (Iuga)/international Continence society (ICS) joint report on the terminology for the conservative and nonpharmacological management of female pelvic floor dysfunction. Int Urogynecol J. 2017;28(2):191–213. 10.1007/s00192-016-3123-4. 27921161

[7] Burgio KL, Newman DK, Rosenberg MT, Sampselle C. Impact of behaviour and lifestyle on bladder health. Int J Clin Pract. 2013;67(6):495–504. 10.1111/ijcp.12143. 23679903

[8] Pinto AM, Subak LL, Nakagawa S, Vittinghoff E, Wing RR, Kusek JW, et al. The effect of weight loss on changes in health-related quality of life among overweight and obese women with urinary incontinence. Qual Life Res. 2012;21(10):1685–94. 10.1007/s11136-011-0086-2. 22161726 PMC3375350

[9] Sigurdardottir T, Steingrimsdottir T, Geirsson RT, Halldorsson TI, Aspelund T, Bø K. Can postpartum pelvic floor muscle training reduce urinary and anal incontinence?: An assessor-blinded randomized controlled trial. Am J Obstet Gynecol. 2020;222(3):.e247.1–78. 10.1016/j.ajog.2019.09.011. 31526791

[10] Wood LN, Markowitz MA, Parameshwar PS, Hannemann AJ, Ogawa SL, Anger JT, et al. Is it safe to reduce water intake in the overactive bladder population? A systematic review. J Urol. 2018;200(2):375–81. 10.1016/j.juro.2018.02.3089. 29499207

[11] Vaughan CP, Markland AD. Urinary incontinence in women. Ann Intern Med. 2020;172(3):ITC17–32. 10.7326/AITC202002040. 32016335

[12] de Viñaspre Rr, Garrido AE, Alvarez MA, de Viñaspre RR. Training women’s pelvic floor muscles during pregnancy and postpartum at primary health centers: a best practice implementation project. JBI Evidence Implementation. 2021;19(3):245–56. 10.1097/XEB.0000000000000288. 34224524

[13] Dicenso A, Bayley L, Haynes RB. Accessing pre-appraised evidence: fine-tuning the 5S model into a 6S model. Evid Based Nurs. 2009;12(4):99.2–101. 10.1136/ebn.12.4.99-b. 19779069

[14] Gavriilidis P, Askari A, Roberts KJ, Sutcliffe RP. Appraisal of the current guidelines for management of cholangiocarcinoma—using the Appraisal of guidelines research and evaluation II (AGREE II) instrument. Hepatobiliary Surg Nutr. 2020;9(2):126–35. 10.21037/hbsn.2019.09.06. 32355672 PMC7188526

[15] Joanna Briggs Institute. The Joanna Briggs Institute Critical Appraisal Tools. 2020. https://joannabriggs.org/critical-appraisal-tools.

[16] Wang Q, Hu Y. JBI evidence pre-grading and evidence recommendation level system (2014 edition). J Educ Chang Nurses Train. 2015;30:964–67.

[17] Davila GW, Martin L. Urinary incontinence in women. 2020. https://bestpractice.bmj.com/topics/en-gb/169.

[18] Lukacz ES. Female urinary incontinence: evaluation. 2022. https://www.uptodate.cn/contents/female-urinary-incontinence-evaluation.

[19] Lukacz ES. Female urinary incontinence: treatment. 2022. https://www.uptodate.cn/contents/female-urinary-incontinence-treatment.

[20] Crowley K, Martin KA, Armsby C, et al. Patient education: urinary incontinence in females (the basics). 2022. https://www.uptodate.cn/contents/urinary-incontinence-in-females-the-basics.

[21] Lukacz ES. Patient education: urinary incontinence treatments for women (beyond the basics). 2022. https://www.uptodate.cn/contents/urinary-incontinence-treatments-for-women-beyond-the-basics?search.

[22] JBI. Recommended practice. Urinary incontinence (older people): management by personal care workers. The JBI EBP Database. 2021;JBI-RP-4611–2.

[23] JBI. Recommended practice. Urinary incontinence in women: prenatal pelvic floor muscle training. The JBI EBP Database. 2022;JBI-RP-4514–2.

[24] JBI. Recommended practice. Urinary incontinence: conservative management. The JBI EBP Database. 2021;JBI-RP-4612–2.

[25] Jayasekara R. Evidence summary. Urinary incontinence: prompted voiding. The JBI EBP Database. 2022;JBI-ES-3499–3.

[26] Moola S, Aginga C. Evidence summary. Stress and urge urinary incontinence in women: pelvic floor muscle training. The JBI EBP Database. 2022;JBI-ES-2576–2.

[27] Koh G. Evidence summary. Urinary incontinence: timed voiding. The JBI EBP Database. 2022;JBI-ES-2082–3.

[28] Porritt K, Abdulsalam A. Evidence summary. Pelvic floor muscle training (antenatal and post-natal): prevention and treatment of urinary incontinence. The JBI EBP Database. 2021;JBI-ES-4002–4.

[29] Mathew S. Evidence summary. Urinary incontinence: treatments. The JBI EBP Database. 2021;JBI-ES-2554–1.

[30] Magtoto L. Evidence summary. Urinary incontinence: conservative management. The JBI EBP Database. 2021;JBI-ES-3190–2.

[31] Aginga C. Evidence summary. Urinary incontinence: assessment. The JBI EBP Database. 2021;JBI-ES-2976–3.

[32] Lizarondo L. Evidence summary. Urinary incontinence in older adults: pelvic floor muscle training. The JBI EBP Database. 2021;JBI-ES-4168–3. 10.1097/XEB.0000000000000432 38747239

[33] Fong E. Evidence summary. Urinary incontinence (older person): assessment. The JBI EBP Database. 2021;JBI-ES-1132–1.

[34] Wang X, Zhang L, Wang Q, Tang A, Xu F. Summary of the best evidence for self-management in patients with urinary incontinence. China Med Her. 2022;19(3):71–74.

[35] Harding CK, Lapitan MC, Arlandis S, et al. Management of non-neurogenic female lower urinary tract symptoms (LUTS). Guidelines The Eur Assoc Urology. 2022.

[36] Registered Nurses’ Association of Ontario C. A proactive approach to bladder and bowel management in adults. 2020.

[37] Dufour S, Wu WM. No. 397 – conservative care of urinary incontinence in women. J Obstet Gynaecol Canada. 2020;42(4):510–22. 10.1016/j.jogc.2019.04.009. 32303295

[38] National Institute for Health and Care Excellence. Pelvic floor dysfunction: prevention and non-surgical management. 2021. https://www.nice.org.uk/guidance/ng210/. 35438877

[39] National Institute for Health and Care Excellence. Urinary incontinence and pelvic organ prolapse in women. Management. 2019. https://www.nice.org.uk/guidance/ng123/. 31211537

[40] Stangel-Wojcikiewicz K, Rogowski A, Rechberger T, et al. Urogynecology Section of the Polish society of Gynecologists and Obstetricians guidelines on the management of stress urinary incontinence in women. Ginekol Pol. 2021;92(11):822–28. 10.5603/GP.a2021.0206. 34907521

[41] Abrams P, Cardozo L, Wagg A, et al. Incontinence. 6th. Vol. 2017. International Continence Society; 2017.

[42] Rawin M, Jones FR, Pond D, et al. RACGP-aged care clinical guide (Silver Book) part A. 2019.

[43] Gao Y, Hu X, Xu F. Systematic assessment on health education of pelvic floor muscle exercise for female patients with stress urinary incontinence. Shanghai Nurs. 2020;20(4):9–14.

[44] Xia J, Duan X, Yu C, Jie Y, Zhang J, Wang K. Meta analysis of pelvic floor muscle training for improving postpartum stress urinary incontinence. Chongqing Med. 2020;49(22):3817–22.

[45] Chen G, Wu L, Huang L, Ding M, Guo F, Cui F. Meta-analysis of the short- and medium-term efficacy of pelvic floor muscle training to improve urinary incontinence symptoms in women. Chin J Phys Med Rehabil. 2020;42(3):265–70.

[46] Wang X, Duan P, Du S, Zhang Q, Guo. Meta-analysis of effect of pelvic floor muscle training during pregnancy to prevent or treat urinary incontinence in primipara. Chin Nurs Res. 2019;33(1):29–36.

[47] Dumoulin C, Cacciari LP, Hay-Smith EJC. Pelvic floor muscle training versus no treatment, or inactive control treatments, for urinary incontinence in women. Cochrane Database Systematic Rev. 2018;2018(10): CD005654. 10.1002/14651858.CD005654.pub4. PMC6516955 30288727

[48] Radzimińska A, Strączyńska A, Weber-Rajek M, Styczyńska H, Strojek K, Piekorz Z. The impact of pelvic floor muscle training on the quality of life of women with urinary incontinence: a systematic literature review. CIA. 2018;13:957–65. 10.2147/CIA.S160057. PMC5962309 29844662

[49] Nipa S, Sriboonreung T, Paungmali A, Phongnarisorn C. Effectiveness of therapeutic interventions for women with urinary incontinence: a systematic review. Crit Rev Phys Rehabil Med. 2020;32(1):1–22. 10.1615/CritRevPhysRehabilMed.2020031380.

[50] García-Sánchez E, Ávila-Gandía V, López-Román J, Martínez-Rodríguez A, Rubio-Arias JÁ. What pelvic floor muscle training load is optimal in minimizing urine loss in women with stress urinary incontinence? A systematic review and meta-analysis. IJERPH. 2019;16(22):4358. 10.3390/ijerph16224358. 31717291 PMC6887794

[51] Nie XF, Ouyang YQ, Wang L, Redding SR. A meta-analysis of pelvic floor muscle training for the treatment of urinary incontinence. Intl J Gynecology Obste. 2017;138(3):250–55. 10.1002/ijgo.12232. 28602038

[52] Mazur-Bialy AI, Kołomańska-Bogucka D, Nowakowski C, Tim S. Urinary incontinence in women: modern methods of physiotherapy as a support for surgical treatment or independent therapy. JCM. 2020;9(4):1211. 10.3390/jcm9041211. 32340194 PMC7230757

[53] López-Liria R, Varverde-Martínez MDLÁ, Padilla-Góngora D, Rocamora-Pérez P. Effectiveness of physiotherapy treatment for urinary incontinence in women: a systematic review. J Retailing Women’s Health. 2019;28(4):490–501. 10.1089/jwh.2018.7140. 30575448

[54] Paiva LL, Ferla L, Darski C, Catarino BM, Ramos JGL. Pelvic floor muscle training in groups versus individual or home treatment of women with urinary incontinence: systematic review and meta-analysis. Int Urogynecol J. 2017;28(3):351–59. 10.1007/s00192-016-3133-2. 27613622

[55] Yang X, Zhang A, Sayer L, Bassett S, Woodward S. The effectiveness of group-based pelvic floor muscle training in preventing and treating urinary incontinence for antenatal and postnatal women: a systematic review. Int Urogynecol J. 2022;33(6):1407–20. 10.1007/s00192-021-04960-2. 34453550 PMC9206632

[56] Malinauskas AP, Bressan EFM, de Melo A, Brasil CA, Lordêlo P, Torelli L. Efficacy of pelvic floor physiotherapy intervention for stress urinary incontinence in postmenopausal women: systematic review. Arch Gynecol Obstet. 2022;308(1):13–24. 10.1007/s00404-022-06693-z. 35831758

[57] Todhunter-Brown A, Hazelton C, Campbell P, Elders A, Hagen S, McClurg D. Conservative interventions for treating urinary incontinence in women: an overview of cochrane systematic reviews. Cochrane Database Systematic Rev. 2022;2022(9): CD012337. 10.1002/14651858.CD012337.pub2. PMC9437962 36053030

[58] Wieland LS, Shrestha N, Lassi ZS, Panda S, Chiaramonte D, Skoetz N. Yoga for treating urinary incontinence in women. Cochrane Database Systematic Rev. 2019;2019(2): CD012668. 10.1002/14651858.CD012668.pub2. PMC6394377 30816997

[59] Woodley SJ, Lawrenson P, Boyle R, Cody JD, Mørkved S, Kernohan A, et al. Pelvic floor muscle training for preventing and treating urinary and faecal incontinence in antenatal and postnatal women. Cochrane Database Syst Rev. 2020;5(5): CD007471. 10.1002/14651858.CD007471.pub4. PMC7203602 32378735

[60] Zhang L, Ren P, Lin M, Hu X, Tang A, Zhang Y, et al. Meta analysis of the effect of self-management intervention in elderly patients with urinary incontinence. China Med Her. 2021;18(33):151–55.

[61] Bordeianou LG, Anger JT, Boutros M, Birnbaum E, Carmichael JC, Connell KA, et al. Measuring pelvic floor disorder symptoms using patient-reported instruments: proceedings of the consensus meeting of the pelvic floor consortium of the American society of colon and rectal surgeons, the international continence society, the American urogynecologic society, and the society of urodynamics, female pelvic medicine and urogenital reconstruction. Female Pelvic Med Reconstruct Surg. 2020;26(1):1–15. 10.1097/SPV.0000000000000817. 31833996

[62] Song X, Bai W, Zhu L, Wang J, Liu P, Tan S, et al. Chinese expert consensus on weight management in obese female patients with stress urinary incontinence. J Clin Med Pract. 2020;24(2):1–5.

[63] Gynecological Pelvic Floor Group of the Obstetrics and Gynecology Branch of the Chinese Medical Association. Guidelines for the diagnosis and treatment of female stress urinary incontinence (2017). Chin J Obstet Gynecology. 2017;52(5):289–93.

[64] Russo E, Caretto M, Giannini A, Bitzer J, Cano A, Ceausu I, et al. Management of urinary incontinence in postmenopausal women: an EMAS clinical guide. Maturitas. 2021;143:223–30. 10.1016/j.maturitas.2020.09.005. 33008675

[65] Hoedl M, Schoberer D, Halfens RJG, Lohrmann C. Adaptation of evidence-based guideline recommendations to address urinary incontinence in nursing home residents according to the ADAPTE-process. J Clin Nurs. 2018;27(15–16):2974–83. 10.1111/jocn.14501. 29700878

[66] Hay-Smith EJC, Herderschee R, Dumoulin C, Herbison GP. Comparisons of approaches to pelvic floor muscle training for urinary incontinence in women. Cochrane Database Systematic Rev. 2011;12): CD009508. 10.1002/14651858.CD009508. 22161451

[67] de Oliveira Camargo F, Rodrigues AM, Arruda RM, Ferreira Sartori MG, Girão, Castro RA, et al. Pelvic floor muscle training in female stress urinary incontinence: comparison between group training and individual treatment using PERFECT assessment scheme. Int Urogynecol J Pelvic Floor Dysfunct. 2009;20(12):1455–62. 10.1007/s00192-009-0971-1. 19690792

[68] Lamb SE, Pepper J, Lall R, Jørstad-Stein EC, Clark MD, Hill L, et al. Group treatments for sensitive health care problems: a randomised controlled trial of group versus individual physiotherapy sessions for female urinary incontinence. BMC Womens Health. 2009;9:1–9. 10.1186/1472-6874-9-26. 19751517 PMC2754423

[69] Hong X, Zhang S, Li G, Jiang B, Chen G. Influence of pelvic floor muscle training in late pregnancy of umbilical artery blood gas analysis index and neonatal asphyxia. China Med Pharm. 2016;6(17):138–41.

[70] Raul A. Exercise during pregnancy and the postpartum period. https://www.uptodate.cn/contents/exercise-during-pregnancy-and-the-postpartum-period. 10.1097/00003081-200306000-00028 12808399

[71] Kincade JE, Dougherty MC, Carlson JR, Hunter GS, Busby-Whitehead J. Randomized clinical trial of efficacy of self-monitoring techniques to treat urinary incontinence in women. Neurourol Urodyn. 2007;26(4):507–11. 10.1002/nau.20413. 17366526

[72] Gleason JL, Richter HE, Redden DT, Goode PS, Burgio KL, Markland AD. Caffeine and urinary incontinence in US women. Int Urogynecol J. 2013;24(2):295–302. 10.1007/s00192-012-1829-5. 22699886 PMC3505252

[73] Baek JM, Song JY, Lee SJ, Park EK, Jeung IC, Kim CJ, et al. Caffeine intake is associated with urinary incontinence in Korean postmenopausal women: results from the Korean national health and nutrition examination survey. PLoS One. 2016;11(2):e0149311. 10.1371/journal.pone.0149311. PMC4762759 26901426

[74] Committee on practice bulletins - Gynecology and the American urogynecologic society. ACOG practice bulletin no. 155: urinary incontinence in women. Obstet Gynecol. 2015;126(5):e66–81. 10.1097/AOG.0000000000001148. 26488524

[75] Auwad W, Steggles P, Bombieri L, Waterfield M, Wilkin T, Freeman R. Moderate weight loss in obese women with urinary incontinence: a prospective longitudinal study. Int Urogynecol J. 2008;19(9):1251–59. 10.1007/s00192-008-0616-9. 18421406

[76] Subak LL, Wing R, West DS, Franklin F, Vittinghoff E, Creasman JM, et al. Weight loss to treat urinary incontinence in overweight and obese women. N Engl J Med. 2009;360(5):481–90. 10.1056/NEJMoa0806375. 19179316 PMC2877497

[77] Danforth KN, Shah AD, Townsend MK, Lifford KL, Curhan GC, Resnick NM, et al. Physical activity and urinary incontinence among healthy, older women. Obstet Gynecol. 2007;109(3):721–27. 10.1097/01.AOG.0000255973.92450.24. 17329526

[78] Townsend MK, Danforth KN, Rosner B, Curhan GC, Resnick NM, Grodstein F. Physical activity and incident urinary incontinence in middle-aged women. J Urol. 2008;179(3):1012–17. 10.1016/j.juro.2007.10.058. 18206951 PMC2712871

[79] Perera J, Kirthinanda DS, Wijeratne S, Wickramarachchi TK. Descriptive cross sectional study on prevalence, perceptions, predisposing factors and health seeking behaviour of women with stress urinary incontinence. BMC Women’s Health. 2014;14(1):78. 10.1186/1472-6874-14-78. 24985068 PMC4094634

[80] Kinchen KS, Burgio K, Diokno AC, Fultz NH, Bump R, Obenchain R. Factors associated with women’s decisions to seek treatment for urinary incontinence. J Retailing Women’s Health. 2003;12(7):687–98. 10.1089/154099903322404339. 14583109

[81] Pakgohar M, Sabetghadam S, Vasegh Rahimparvar SF, Kazemnejad A. Quality of life (QoL) and help-seeking in postmenopausal women with urinary incontinence (UI): a population based study. Arch Gerontol Geriat. 2014;59(2):403–07. 10.1016/j.archger.2014.07.004. 25067833

[82] Ho L, Macnab A, Matsubara Y, Peterson K, Tsang B, Stothers L. Rating of pelvic floor muscle training mobile applications for treatment of urinary incontinence in women. Urology. 2021;150:92–98. 10.1016/j.urology.2020.08.040. 32890617

