# Supplementary material for: Pelvic floor rehabilitation management in females with stress urinary incontinence: a summary of best-evidence strategies
Source: BMC Nurs. 2026 Jun 21;25:790. doi: 10.1186/s12912-026-04874-8 (PMC13536786; doi:10.1186/s12912-026-04874-8)
Supplement: Supplementary file 1 [file 12912_2026_4874_MOESM1_ESM.docx]

**Appendix 1 search strategy**

**Database: PubMed**

**Results: 1282**

**Search Period：1950** **to September 14, 2022**

| **Number** | **Search Terms** | **Results** |
| --- | --- | --- |
| #1 | urinary incontinence[MeSH Terms] | 35,412 |
| #2 | urinary incontinence[Title/Abstract] | 27,640 |
| #3 | urinary leakage[Title/Abstract] | 1,147 |
| #4 | urinary incontinence, stress[MeSH Terms] | 12,472 |
| #5 | urinary incontinence, stress[Title/Abstract] | 230 |
| #6 | stress incontinence[Title/Abstract] | 5,936 |
| #7 | pelvic floor disorders[MeSH Terms] | 1,154 |
| #8 | pelvic floor disorder*[Title/Abstract] | 1,441 |
| #9 | pelvic floor disease*[Title/Abstract] | 46 |
| #10 | pelvic floor dysfunction*[Title/Abstract] | 1,355 |
| #11 | (((((((((urinary incontinence[MeSH Terms]) OR (urinary incontinence[Title/Abstract])) OR (urinary leakage[Title/Abstract])) OR (urinary incontinence, stress[MeSH Terms])) OR (urinary incontinence, stress[Title/Abstract])) OR (stress incontinence[Title/Abstract])) OR (pelvic floor disorders[MeSH Terms])) OR (pelvic floor disorder*[Title/Abstract])) OR (pelvic floor disease*[Title/Abstract])) OR (pelvic floor dysfunction*[Title/Abstract]) | 47,537 |
| #12 | management[Title/Abstract] | 1,354,839 |
| #13 | health education[MeSH Terms] | 259,327 |
| #14 | health education[Title/Abstract] | 40,074 |
| #15 | pelvic floor muscle training[Title/Abstract] | 1,091 |
| #16 | pelvic floor muscle exercise*[Title/Abstract] | 404 |
| #17 | kegel exercise*[Title/Abstract] | 157 |
| #18 | pelvic floor rehabilitation[Title/Abstract] | 208 |
| #19 | lifestyle intervention*[Title/Abstract] | 9,251 |
| #20 | behavior modification*[Title/Abstract] | 2,557 |
| #21 | self-management[MeSH Terms] | 4,769 |
| #22 | self-management[Title/Abstract] | 24,670 |
| #23 | non-drug treatment[Title/Abstract] | 226 |
| #24 | non-pharmacological treatment[Title/Abstract] | 1,599 |
| #25 | non-drug therap*[Title/Abstract] | 355 |
| #26 | non-surgical treatment[Title/Abstract] | 3,331 |
| #27 | conservative therap*[Title/Abstract] | 9,826 |
| #28 | conservative treatment[MeSH Terms] | 4,700 |
| #29 | conservative treatment[Title/Abstract] | 34,135 |
| #30 | (((((((((((((((((management[Title/Abstract]) OR (health education[MeSH Terms])) OR (health education[Title/Abstract])) OR (pelvic floor muscle training[Title/Abstract])) OR (pelvic floor muscle exercise*[Title/Abstract])) OR (kegel exercise*[Title/Abstract])) OR (pelvic floor rehabilitation[Title/Abstract])) OR (lifestyle intervention*[Title/Abstract])) OR (behavior modification*[Title/Abstract])) OR (self-management[MeSH Terms])) OR (self-management[Title/Abstract])) OR (non-drug treatment[Title/Abstract])) OR (non-pharmacological treatment[Title/Abstract])) OR (non-drug therap*[Title/Abstract])) OR (non-surgical treatment[Title/Abstract])) OR (conservative therap*[Title/Abstract])) OR (conservative treatment[MeSH Terms])) OR (conservative treatment[Title/Abstract]) | 1,656,122 |
| #31 | guideline[MeSH Terms] | 172,555 |
| #32 | guideline*[Title/Abstract] | 441,724 |
| #33 | guidance[Title/Abstract] | 146,295 |
| #34 | clinical practice[Title/Abstract] | 231,798 |
| #35 | best practice[Title/Abstract] | 17,203 |
| #36 | evidence summary[Title/Abstract] | 339 |
| #37 | evidence synthesis[Title/Abstract] | 6,361 |
| #38 | recommendation[Title/Abstract] | 52,963 |
| #39 | consensus[MeSH Terms] | 19,087 |
| #40 | consensus*[Title/Abstract] | 196,482 |
| #41 | systematic review[Publication Type] | 205,922 |
| #42 | meta-analysis[Publication Type] | 166,643 |
| #43 | (((((((((((guideline[MeSH Terms]) OR (guideline*[Title/Abstract])) OR (guidance[Title/Abstract])) OR (clinical practice[Title/Abstract])) OR (best practice[Title/Abstract])) OR (evidence summary[Title/Abstract])) OR (evidence synthesis[Title/Abstract])) OR (recommendation[Title/Abstract])) OR (consensus[MeSH Terms])) OR (consensus*[Title/Abstract])) OR (systematic review[Publication Type])) OR (meta-analysis[Publication Type]) | 1,294,666 |
| #44 | (((((((((((urinary incontinence[MeSH Terms]) OR (urinary incontinence[Title/Abstract])) OR (urinary leakage[Title/Abstract])) OR (urinary incontinence, stress[MeSH Terms])) OR (urinary incontinence, stress[Title/Abstract])) OR (stress incontinence[Title/Abstract])) OR (pelvic floor disorders[MeSH Terms])) OR (pelvic floor disorder*[Title/Abstract])) OR (pelvic floor disease*[Title/Abstract])) OR (pelvic floor dysfunction*[Title/Abstract])) AND ((((((((((((((((((management[Title/Abstract]) OR (health education[MeSH Terms])) OR (health education[Title/Abstract])) OR (pelvic floor muscle training[Title/Abstract])) OR (pelvic floor muscle exercise*[Title/Abstract])) OR (kegel exercise*[Title/Abstract])) OR (pelvic floor rehabilitation[Title/Abstract])) OR (lifestyle intervention*[Title/Abstract])) OR (behavior modification*[Title/Abstract])) OR (self-management[MeSH Terms])) OR (self-management[Title/Abstract])) OR (non-drug treatment[Title/Abstract])) OR (non-pharmacological treatment[Title/Abstract])) OR (non-drug therap*[Title/Abstract])) OR (non-surgical treatment[Title/Abstract])) OR (conservative therap*[Title/Abstract])) OR (conservative treatment[MeSH Terms])) OR (conservative treatment[Title/Abstract]))) AND ((((((((((((guideline[MeSH Terms]) OR (guideline*[Title/Abstract])) OR (guidance[Title/Abstract])) OR (clinical practice[Title/Abstract])) OR (best practice[Title/Abstract])) OR (evidence summary[Title/Abstract])) OR (evidence synthesis[Title/Abstract])) OR (recommendation[Title/Abstract])) OR (consensus[MeSH Terms])) OR (consensus*[Title/Abstract])) OR (systematic review[Publication Type])) OR (meta-analysis[Publication Type])) | 1,282 |
